# Progreso en Salud: Findings from Two Adapted Social Network HIV Risk Reduction Interventions for Latina Seasonal Workers

**DOI:** 10.3390/ijerph16224530

**Published:** 2019-11-15

**Authors:** Mariano Kanamori, Mario De La Rosa, Cho-Hee Shrader, Cesar Munayco, Susanne Doblecki-Lewis, Guillermo Prado, Steven Safren, Mary Jo Trepka, Kayo Fujimoto

**Affiliations:** 1 Department of Public Health Sciences, University of Miami School of Medicine, Miami, FL 33136, USA; cxs939@miami.edu (C.-H.S.); GPrado@med.miami.edu (G.P.); 2 Robert Stempel College of Public Health & Social Work, Florida International University, Miami, FL 33199, USA; Delarosa@fiu.edu (M.D.L.R.); TrepkaM@fiu.edu (M.J.T.); 3 Centro Nacional De Epidemiología, Prevención y Control de Enfermedades, Ministerio de Salud, Lima 15072, PERU; cmunayco@dge.gob.pe; 4 Department of Medicine, University of Miami School of Medicine, Miami, FL 33136, USA; SDoblecki@med.miami.edu; 5 Department of Psychology, University of Miami School of Medicine, Miami, FL 33136, USA; SSafren@miami.edu; 6 Department of Health Promotion & Behavioral Sciences, School of Public Health, University of Texas Health Science Center at Houston, Houston, TX 77030, USA; kayo.fujimoto@uth.tmc.edu

**Keywords:** prevention science, intervention development, social network analysis, Hispanic Americans, HIV/AIDS

## Abstract

Background: Miami-Dade County, where many Latina seasonal workers reside and work, has the highest incidence of the human immunodeficiency virus (HIV) in the US: a rate four times the national average. Despite this disproportionate risk for HIV, there are no HIV prevention interventions that aim to decrease HIV among Latina seasonal workers. Methods: The PROGRESO EN SALUD study compared the outcomes of two interventions adapted to include a social network component (VOICES and HEALTHY). Recruitment used a social network respondent-driven sampling design in which each seed was asked to recruit three friends, and those friends were asked to recruit three friends, for a total of twenty groups of 13 friends. We collected data at baseline, and 6 months and 12 months post intervention completion. We used generalized estimating equation models, properly adjusted for non-independent contributions of both social network interventions, to estimate the effects. Gaussian family multivariate models were calculated, addressing exchangeable working correlations, including both individual-level and cluster-level covariates in these models. Results: A total of 261 Latina seasonal workers participated in either the HEALTHY or the VOICES intervention. There were significant changes over time in cognitive factors (*HIV knowledge, condom use self-efficacy*, and *adequate knowledge of condom use*), behavioral factors (*condom use, female condom use*, and *HIV testing*), and communication factors (*talking with friends about HIV prevention* and *intention to negotiate safe sex with male partners*). Discussion: This study supports the literature suggesting that interventions incorporating social networks can have positive effects on HIV prevention and treatment outcomes, including sustained benefits beyond study periods.

## 1. Introduction

HIV persists as a serious public health issue for Hispanic/Latina women (Latinas) in the US [1,2,3]. The likelihood of being diagnosed with HIV in her lifetime is significantly higher for Latinas (1 in 106) than for non-Latina white women (1 in 526) in the US [1,2,3]. Poverty, unemployment, low education, discrimination, and barriers to healthcare access increase Latinas’ risk for HIV infection [4]. One fourth of Latinas in the US live under the poverty line, and over half are near poverty levels [5]. One in five Latinas ages 25–29 year has not graduated from high school as compared to one in 12 women from all other ethnic/racial groups [6]. One of the most disadvantaged and underserved groups of adult Latinas are seasonal workers. Latina seasonal workers (LSW) are defined in this study as individuals who work or whose spouse/partner works in agriculture at a location within 75 miles of their home [7,8]. LSW are economically disadvantaged in the US with approximately 75% living below the poverty level [9]. Areas in the South and areas with a high concentration of Latinos, such as South Florida, are significantly impacted by the HIV epidemic [10]. However, there are few studies implementing and evaluating HIV prevention interventions for this at-risk population.

Miami-Dade County (MDC) is the epicenter of the HIV epidemic in the US, with an incidence four times the national average [11]. Among adults diagnosed with HIV in 2017, Latinos accounted for 59% of cases in Miami Dade County [12]. MDC’s HIV epidemic predominantly affects men, with only 26% of people living with HIV being women. However, in Homestead, Florida City, Naranja and vicinity areas, where the majority of residents are LSW, 44% of people living with HIV are female [13]. Further, 85% of women reported heterosexual sex as the mode of HIV transmission [13]. HIV prevention strategies for underserved Latinas, such as seasonal workers, should consider cultural norms, cultural values, and socialization contexts, which influence Latinas’ risk for HIV [14]. Social network-based interventions address these considerations by (1) incorporating cultural norms and values such as *personalismo* (i.e., valuing interpersonal relationships and social interactions) and socialization contexts [15]; (2) using link-tracing designs to recruit from high risk populations, build trust with intervention participants, and increase participation rates; and (3) providing an opportunity for members of disenfranchised communities to serve as leaders, health intervention champions, and positive role models [16]. Despite these advantages of social network-based interventions, there are currently no HIV prevention education programs for LSW that incorporate social network approaches. The present study PROGRESO EN SALUD (PROGRESO), designed to address this need, evaluated two interventions (VOICES and HEALTHY) based on social network recruitment approaches to increase: (1) cognitive factors (i.e., *HIV knowledge* and *condom use self-efficacy*), (2) behavioral factors (i.e., *HIV testing, condom use*, and *female condom use*), and (3) communication factors regarding HIV (i.e., *having conversations with friends about HIV prevention* and *intention to negotiate safe sex with male partner*).

## 2. Materials and Methods

The longitudinal PROGRESO study consisted of a total of three visits over a 12-months period. We recruited and enrolled 261 LSW in Miami Dade County-Florida (Homestead, Florida City, Naranja, and vicinity areas). The first enrollment occurred in June 2015, the last enrollment in April 2016, and the last follow-up in February 2017. Eligibility criteria included: (1) identifying as a female age 18 years or older, (2) living in a seasonal worker community, (3) being able to speak and understand Spanish, (4) reporting at least one episode of condomless sex, (5) reporting consumption of alcohol or other drugs three months prior to the baseline interview, (6) reporting likely to remain in the general geographic area for the next two years, and (7) being able to understand and provide written informed consent. We guided the structure of this paper using the Consolidated Standards of Reporting Trials (CONSORT) 2010 guidelines [17].

Using community-based participatory research approaches, we identified a community partner located in a Latino seasonal working community in South Florida to implement this study. Recruitment and intervention procedures were designed to incorporate Latino cultural values such as *personalismo* and *collectivism*. The cultural value of *personalismo* refers to a preference for friendship with close individuals with similar sociodemographic characteristics (i.e., *homophily*), suggesting a preference for familiarity in these relationships [15]. Thus, *personalismo* was incorporated into the social network recruitment and configuration of the sample in a way that reflected the preference for holding conversations about sensitive topics, such as HIV prevention, only after establishing friendships based on trust, support, and empathy [18,19].

The pre-implementation phases included the following activities in accordance with the Consolidated Framework for Implementation Research domains: revising the intervention kit material; assessing the available resources and cost of implementation; developing the program budget; identifying appropriate setting, space, and equipment with the collaborating community partner; assessing staff’s capacity to conduct the intervention; training staff members using the VOICES manual on leading skill-building sessions, condom use, negotiation skills, and evaluation tools for program implementation; identifying and developing adequate and innovative methods to recruit Latina seasonal workers at risk for HIV; identifying and hiring members from the community with adequate skills to perform small group facilitation and recruitment of participants; implementing the human subjects protection training with all personnel involved in the program; establishing recruitment partnerships with over 30 local/state health officials and private and public organizations working with the seasonal worker community; implementing a qualitative study (3 focus groups; *N* = 29 Latina seasonal workers) to gain specific information regarding this community; and developing an adaptation of the intervention implementation using a community-based participatory research approach [20]. Our partnering community-based organization played a key role in all phases of the study, including delivery of the intervention and data collection. Study participants were recruited at locations where seasonal workers are known to reside, including trailer parks, dormitory-style housing, apartment buildings, motels, duplexes, neighborhoods of single/duplex housing, and seasonal workplaces in the Homestead/Florida City area (Miami-Dade County).

Members from our community partner identified twenty seed individuals, the “Latina leaders”. Seeds were respected women in the seasonal worker community who had the ability to reach many peers. The first ten enrolled seeds were assigned to VOICES and the last ten seeds to HEALTHY. Recruitment procedures incorporated the cultural value of *collectivism.* In Latino communities, *collectivism* is a cultural orientation that values close, nurturing, and supportive interpersonal relationships over individualistic behaviors and attitudes. According to this orientation, a person is seen as part of a family and broader community of friends and acquaintances and should accept responsibility for this role [21]. Consistent with *collectivism* values, in both interventions, we provided Latina leader seeds with instructions on how to promote interactions and conversations about HIV prevention within their social networks, so that underserved Latinas in their community could support one another.

We implemented a link-tracing or social network respondent-driven sampling design to configure groups of 13 participants, an adequate number for a social network-based intervention. Each seed was asked to invite three friends, (i.e., first-order friends). The first-order friends were further asked to invite three of their own friends (i.e., second-order friends) [22]. As a result, for each seed, a network of 13 actors was recruited in three respondent-driven waves, with wave one including the seed, wave two including three first-order friends, and wave three including nine second-order friends. All participants in the seeds’ chain received the same intervention. Seeds were asked to have all members of their social network contact the project coordinator. If any invited friend declined participation, the seed/friend was asked to invite a substitute friend until a social network of 13 actors was formed. Members from our community partner checked that participants were not part of two social networks. These types of word-of-mouth recruitment strategies incorporating Latino cultural values have been shown to successfully work in similar populations and settings, and they differed from recruitment strategies of the original VOICES intervention, which recruited participants from waiting rooms of HIV and sexually transmitted infections (STI) clinics [23].

### 2.1. The Adapted VOICES Intervention

VOICES is a video-based intervention that is grounded in the Theory of Reasoned Action [24]. According to the Theory of Reasoned Action, human behaviors are guided by two factors: (1) a person’s attitudes, beliefs, and experiences; and (2) how a person believes others think he or she should act in a given circumstance (i.e., the social and cultural norms that are prevalent in that person’s community). VOICES is a US Centers for Disease Control and Prevention (CDC) High Impact Prevention Program (HIP; also known as Diffusion of Effective Behavioral Intervention) which helps bridge the gap between HIV/STI prevention research and practice [25,26,27,28,29,30]. The original VOICES is a single session, video-based program for the prevention of HIV and other sexually transmitted diseases. VOICES was designed to encourage condom use and improve condom negotiation skills among heterosexual African American and Latino men and women, aged 18 years and older, who are at very high risk for HIV and other STIs [25,26,27,28,29,30]. VOICES has been adapted to specific populations such as individuals in correctional settings and men who have sex with men [26], but has not been previously adapted to Latina seasonal workers. In our adapted version of VOICES, we provided Latina Leaders with additional instructions on how to promote interactions and conversations about HIV prevention within their friendship networks. At time points of three months and nine months after the intervention session, Latina Leaders met with the individuals from their network to discuss HIV/STI prevention, either as a group, in a single session, or via individual home visits depending on the participants’ availability. The Latina Leaders were given three HIV/STI informational pamphlets in Spanish to guide conversations each time. These pamphlets were also distributed to each member of her social network. A detailed description of the cultural adaption and the integration of social networks approaches in VOICES is described in Table S1, and additional information can be found elsewhere [7,31].

### 2.2. The HEALTHY Intervention

The HEALTHY intervention aimed to increase participants’ awareness of HIV-specific health issues. Teaching topics included general HIV prevention and risk reduction strategies such as condom use, avoidance of over-the-counter medication, and HIV communication. In addition, Lay Health Advisors spoke about other general health strategies such as hygiene and healthy living in crowded conditions. HEALTHY was similar to VOICES in dosage, as it consisted of one session that lasted approximately three hours. Moreover, similar quality control provisions were used for the HEALTHY condition, particularly regarding fidelity. HEALTHY did not incorporate a video. We developed, culturally adapted, and pre-tested flip-chart posters with large graphical representations used by our Project Coordinator and Lay Health Advisor to provide information and promote discussion surrounding health topics. Seeds were neither asked nor discouraged from promoting conversations around HIV prevention with others in their social networks.

### 2.3. Assessments

At baseline, participants completed a structured individual face-to-face interview (approximately 1.5 h in duration) in Spanish using computer assisted personal interviewing software in a private office of the collaborating community agency. Interviews were performed by six trained bilingual Latinas. The questionnaire was originally developed in English then translated into Spanish and back-translated to English to ensure the integrity of the instrument and language translation competencies. The instrument was pilot tested twice with a total of 10 participants to confirm that the content and language would be clear to participants. Our Lay Health Advisor performed retention activities via phone calls and home visits. Follow-up assessment interviews were similar to baseline assessments and were repeated at 6 months and 12 months post-intervention. These assessments collected information on (1) cognitive factors (i.e., *HIV knowledge* and *condom use self-efficacy*), (2) behavioral factors (i.e., *HIV testing, condom use*, and *female condom use*), and (3) communication factors regarding HIV (i.e., *having conversations with friends about HIV prevention* and *intention to negotiate safe sex with male partner*).

Interviewers discussed the importance of informed consent and obtained written consent from all participants. Both interventions included one health promotion session that lasted approximately three hours. Participant incentives were the following: a bag lunch and $40 for the baseline interview; $40 for attending the intervention; $50 for taking the 6-months interview; and $55 for taking the 12-months interview. In addition, seed individuals received $10 for each participant they referred and who participated in the interviews, $10 for each member of their group who attended the intervention, and $10 for each group member with whom they discussed the informational materials at three- and nine-months post intervention (VOICES). The interventions were audio-recorded to assess the fidelity of the implementation. This study was approved by the Institutional Review Board of Florida International University, and study staff received informed consent from participants prior to data collection.

### 2.4. Measures

*Condom use* was assessed with four items regarding the frequency of condom use during vaginal and anal sex in the past 30 days, with a (a) primary sex partner and/or (b) casual sexual partner (e.g., “How often was a condom used for vaginal sex with your primary sex partner during the past 30 days?”). Questions were measured on a scale from 1 (“always”) to 4 (“never”). The four questions about frequency of condom use (i.e., vaginal sex with primary partner, vaginal sex with casual partner, anal sex with primary partner, and anal sex with casual partner) were combined into a single ordinal variable indicating the least frequent level of condom use with any partner. For example, if the participant reported “never” using a condom for vaginal or anal sex with a primary sex partner and “sometimes” using a condom for vaginal or anal sex with a casual sex partner, that person’s condom use was coded as “never”.

*Female condom use* was assessed by asking, “Have you used female condoms?” (binary).

*HIV testing* was assessed by asking, “Have you been tested for HIV during the past 6 months?” Reports of HIV testing at the 6-months and 12-months follow-up interviews were combined into a single binary variable to assess HIV testing any time during the 12-months post intervention.

*Talk with friends about HIV prevention* was assessed with the following two questions: “Have you talked with your friends about ways to convince your partner to use condoms?” and “Have you talked with your friends about ways to have sex with a condom?” Response options ranged on a five-point scale from 4 (“very often”) to 0 (“never”). The total score was calculated by summing the scores from the two items (range = 2–10), with a higher score representing a higher frequency of communication with friends regarding HIV prevention.

*HIV knowledge* was assessed with 18 true/false items from the validated HIV Knowledge Questionnaire (HIV/-KQ-18) (e.g., “coughing and sneezing do not spread HIV”) [14]. Assigning 1 point to each correct answer, the total score indicated the number of items answered correctly (range = 0–18). A higher score indicated a higher level of HIV-related knowledge.

*Participants’ condom use self-efficacy* was assessed with a seven-item four-point Likert-type scale [32]. Items evaluated self-efficacy by measuring respondents’ agreement with statements such as, “It would be easy to make my partner use a condom”, with possible responses ranging from 1 (“strongly disagree”) to 4 (“strongly agree”). The score from items with statements that did not reflect positive condom use self-efficacy were reversed. The total score was calculated by summing the scores from the seven items (range = 7–28), with a higher score representing a higher level of self-efficacy for HIV prevention strategies. Further information about this measure can be found elsewhere [7].

*Adequate knowledge of condom use* was measured using a five item, five-point Likert-type scale including items such as “You remember to hold the base of the condom when you take the condom off (your partner)”, with responses ranging from 0 (“never”) to 4 (“very often”). The total score was calculated by summing the scores from the five items (range = 0–20), with a higher score representing a higher level of correct use of condoms [7,31].

*Intentions to negotiate safe sex with male partner* was measured with four items (e.g., “I will say no to sex with a male partner if he will not use a condom”). Response options ranged on a four-point scale from 4 (“strongly disagree”) to 1 (“strongly agree”). The total score was calculated by summing the scores from the four items (range = 4–16), with a higher score representing a higher intention to negotiate safe sex with male partner.

### 2.5. Data Analysis

Descriptive statistics at baseline included proportions for categorical variables, and means and standard deviations for continuous variables. Socio-demographic differences between participants who completed the study and those who were lost to follow-up were assessed using chi-square and one-way analysis of variance (ANOVA) tests. Analyses were performed by intention to treat. Because each participant contributed three values (baseline, 6 months, and 12 months), generalized estimating equations (GEE) models were used to estimate the effects, properly adjusted for non-independent contributions of both social network interventions [33,34]. The GEE method is a robust and extensively used method that assesses the time trend in repeated measurements, addressing random, missing, and misspecification of the true correlation structure. Gaussian family multivariate models were calculated addressing exchangeable working correlations with the package *geepack* [34]. Both individual-level and cluster-level covariates were included in these models. In order to address multiple comparison bias, separate models were performed for each outcome. Models were controlled for variable time. We estimated beta coefficients, *p* values, and 95% confidence intervals. Effect sizes were assessed using the R^2^. An α = 0.05 was used throughout the analysis. Analyses were performed using the R software environment.

## 3. Results

A total of 192 participants (74%) completed the 6-months follow-up assessment, and 197 participants (76%) completed the 12-months follow-up assessment. Sample demographics, presented in Table 1, indicate that there were no significant differences between participants from VOICES and HEALTHY.

*Loss to follow-up*. There were no significant differences between participants who completed the 12-months survey and those who did not in terms of age (F_(1)_ = 0.012, *p* = 0.914), education (X^2^_(6)_ = 3.84, *p* = 0.698), marital status (X^2^_(5)_ = 3.90, *p* = 0.564), country of origin (X^2^_(1)_ = 1.21, *p* = 0.271), income (F_(1)_ = 0.049, *p* = 0.825), or having health insurance (X^2^_(1)_ = 3.70, *p* = 0.055) (Results not shown in a table).

### 3.1. Cognitive Factors

*HIV knowledge*. Generalized estimating equation models showed that the *HIV knowledge* trajectory improved significantly between baseline and the 6-months follow-up (*p* = 0.001) and between baseline and the 6-months and 12-months follow-ups (*p* = 0.001) (Table 2). Results also showed that the *HIV knowledge* trajectories were statistically significantly higher for HEALTHY than VOICES (*p* = 0.001). *Condom use self-efficacy* trajectory also improved significantly between baseline and the 6-months follow-up (*p* = 0.05) and between baseline and the 6-months and 12-months follow-ups (*p* = 0.05). *Condom use self-efficacy* trajectories were statistically significantly higher for HEALTHY than VOICES interventions (*p* = 0.05). The trajectory for *adequate condom use knowledge* improved significantly between baseline and the 6-months follow-up (*p* = 0.05) and between baseline and the 6-months and 12-months follow-ups (*p* = 0.001). We found no statistically significant intervention effects for *adequate condom use knowledge*.

### 3.2. Behavioral Factors

**Figure 1:**
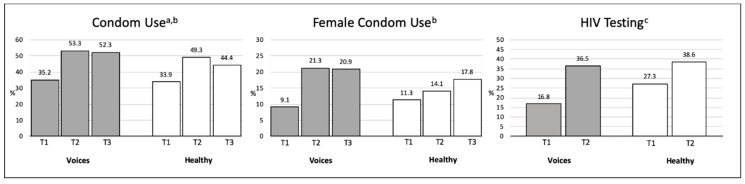
Changes over time in Latinas’ condom use, female condom use, and HIV Testing, South Florida, 2015–2017. ^a^ Condom use was assessed with four items regarding the frequency of vaginal and anal sex with primary sex partner and vaginal or anal sex with a casual sexual partner in the past 30 days. ^b^ Comparison of baseline, 6-months, and 12-months follow-ups. ^c^ HIV testing during the past 6 months. Comparison of baseline and combined answers from 6-months and 12-months follow-ups.

Changes before this program (baseline data) and during the implementation of the program (6-months and 12-months follow-ups) were calculated for *condom use, female condom use, and HIV testing* (Figure 1; Table 2). A significant *condom use* trajectory was found between baseline and the 6-months follow-up (*p* = 0.01) and between baseline and the 6-months and 12-months follow-ups (*p* = 0.01). At baseline, 35% of VOICES participants reported *using a condom during sexual intercourse in the past 30 days* compared to 53% at the 6-months follow-up and 52% at the 12-months follow-up. At baseline, 34% of HEALTHY participants reported *using a condom during sexual intercourse in the past 30 days* compared to 49% at the 6-months follow-up and 44% at the 12-months follow-up (Figure 1). The trajectory of *female condom use* was significant between baseline and the 6-months follow-up (*p* = 0.01) and between baseline and the 6-months and 12-months follow-ups (*p* = 0.01). At baseline, 9% of VOICES participants reported *using a female condom* compared to 52% at the 6-months and 12-months follow-ups. At baseline, 11% of HEALTHY participants reported *using a female condom* compared to 14% at the 6-months follow-up and 18% at the 12-months follow-up. We compared *HIV testing* at baseline and combined answers from the 6-months and 12-months follow-ups. A significant change was found for *HIV testing* (*p* = 0.001; VOICES from 17% to 37%; HEALTHY from 27% to 39%). We found no statistically significant intervention effects for any of the three behavioral factors.

### 3.3. Communication Factors

The trajectory of *having conversations with friends about HIV prevention* was significant between baseline and the 6-months follow-up (*p* = 0.001) and between baseline and the 6-months and 12-months follow-ups (*p* = 0.001) (Table 2). The trajectory for *intention to negotiate safe sex with male partner* was only significant between baseline and the 6-months follow-up (*p* = 0.05). We found no statistically significant intervention effects for these two communication factors.

## 4. Discussion

This study aimed to evaluate two social network-adapted HIV interventions. The 261 LSW participants were recruited through friendship networks and received either the social network adapted VOICES or HEALTHY intervention. Both interventions led to significant changes over time in cognitive factors (*HIV knowledge, condom use self-efficacy*, and *adequate knowledge of condom use*), behavioral factors (*condom use, female condom use*, and *HIV testing*), and communication factors (*talking with friends about HIV prevention* and *intention to negotiate safe sex with male partner*). Overall, both interventions were equally effective with the exception that HEALTHY had a higher effect on *HIV knowledge* and *condom use self-efficacy*.

Our findings strengthen the evidence base supporting the utility of link-tracing recruitment designs to recruit from previously unengaged (“hard-to-reach”) populations. Link-tracing recruitment designs have positive effects on HIV prevention outcomes, including sustained benefits 12-months after the implementation of the intervention session. Our results suggest that existing best practice interventions widely used to address health care concerns affecting Latinas can be enhanced by incorporating social network recruitment approaches to improve cognitive, behavioral, and communication factors. This study also supports the literature suggesting that interventions incorporating social networks can have positive effects on HIV prevention and treatment outcomes, including sustained benefits beyond study periods [35]. Knowledge and behaviors can spread through social ties and interactions via several different mechanisms such as persuasion, communication of norms, modeling, information, support, and social pressure [36,37]. Social networks can amplify diffusion beyond single dyads or pairs of individuals [38,39,40]. As a result, changes in the cognitive factors, behavioral factors, and communication factors of one network “actor” can cascade within and between social networks, producing behavioral changes in the population-at-large. Such cascades offer the prospect of increasing the cost-effectiveness and reach of public health programs that incorporate social network data and analyses, especially in low-resource settings [41,42].

We believe the network-based approach, which incorporated Latino cultural values, was instrumental in the success of both interventions. The incorporation of *personalismo* in using a link-tracing design recruitment allowed for the recruitment of groups of underserved Latina participants who were friends or who had a friend in common. The incorporation of this social network recruitment strategy promoted participants’ engagement. Participants from both intervention groups reported that they communicated among themselves via phone calls to remind each other about attending the intervention or scheduling an interview, and in some cases when a seed could not personally contact one member of her network, contact was made by their mutual friends, usually via social media. Participants reported being interested in participating in these interventions not only for their personal benefit, but also to allow their friends to benefit from the intervention. For instance, some participants gave their program incentives to other participants who could not afford transportation to the intervention. Our social network-based approach, incorporating Latino cultural values, created a social environment that allowed Latina participants to feel comfortable discussing sensitive topics, collaborate among themselves, and develop social capital.

Our findings should be interpreted within the context of the research limitations. First, we cannot attribute with certainty that the differences between interventions is due to the interventions’ effects. The study results could be due to unknown innate differences between the two groups, as a higher proportion of participants in the HEALTHY intervention reported having been tested for HIV at baseline. Nevertheless, the use of GEE models supports the effects of findings being due to the intervention differences [43,44,45]. Second, the study depended on self-reported measures of sensitive information, such as sexual behavior; thus, social desirability (potentially introduced by face-to-face interviews) and recall bias may have exaggerated the effects of both interventions away from the null. However, as these biases were observed in both groups, we believe they are accounted for in our GEE model. Additionally, care was taken to hire experienced Latina interviewers trained in culturally-appropriate interviewing techniques for data collection. Third, given that our interventions were conducted with a unique population of Latinas, future studies that replicate the aforementioned methods in other regions of the United States and/or with different Latino populations may yield different results. Fourth, this study cannot assess whether the VOICES or HEALTHY intervention was better than usual practice. An important limitation is that we did not include a usual practice control because the study focused on understanding whether it was feasible to implement social network-based interventions in the LSW community and assessing the potential efficacy of these interventions.

## 5. Conclusions

Future adaptations of HIV-prevention interventions, including VOICES and HEALTHY should also incorporate biomedical prevention strategies, such as HIV pre-exposure prophylaxis (PrEP). The United States Food and Drug Administration approved the use of the anti-retroviral combination of tenofovir and emtricitabine for PrEP in July 2012, in tandem with our program’s design [46]. As a result, the present study did not incorporate PrEP education into the intervention components. PrEP has been shown to reduce HIV transmission in sexually active heterosexual adults by up to 60% and for individuals who take PrEP daily, the HIV transmission rate is reduced up to 99% [47,48,49,50]. Many underserved communities are unaware of the existence and/or efficacy of PrEP [51,52,53,54]. Innovative strategies are needed for dissemination of PrEP information to women at risk for HIV in a way that is believable and empowering [54]. In future work, we plan to adapt the social network approach used to include PrEP knowledge and uptake among LSW at risk for HIV infection. Future research is needed to support our promising findings, suggesting that social network methods can be incorporated into effective HIV prevention interventions for LSW and women from other communities.

## Figures and Tables

**Table 1:** Baseline characteristics between participants from VOICES and HEALTHY, South Florida, 2015.

	VOICES		HEALTHY		
Characteristics	*n*	%	*n*	%	*p* Value
Total	127	100	131	100	
*Mean age in years* (standard deviation)	34.8	(9.9)	34.5	(10.3)	0.834
*Highest level of Education*					
None	8	6.3	7	5.3	0.226
From 1st to 8th grade	54	42.5	38	29.0	
9th to 11th grade	19	15.0	23	17.6	
High school graduate or equivalent degree	18	14.2	20	15.3	
Post high school education	18	14.2	21	16.0	
Some Bachelor-level college education	10	16.8	22	16.8	
*Marital Status*					
Single	22	17.3	29	22.1	0.472
Legally married	50	39.4	49	37.4	
Cohabitating	48	37.8	41	31.3	
Separated/divorced	6	4.8	12	9.1	
Widow	1	0.8	0	0.0	
*Country of Birth*					
Non US-born	105	82.7	108	82.4	0.960
US-born	22	17.3	23	17.6	
*Mean total income from the past 6 months*, *US dollars* (standard deviation)	5506	(508)	5611	(492)	0.122
*Has health insurance*					
Yes	27	21.3	32	24.4	0.324
No	100	78.7	99	75.6	

**Table 2:** Intervention effects on cognitive, behavioral, and communication factors.

Trajectories	*b*	SE	95% CI	Effect Size (d)
*Cognitive Factors*				
HIV Knowledge				0.09
6-months follow-up	2.35 ***	0.39	1.59–3.11	
12-months follow-up	2.45 ***	0.41	1.65–3.26	
VOICES vs. HEALTHY	−1.22 ***	0.33	−1.86–0.57	
Condom use self-efficacy				0.02
6-months follow-up	0.84 *	0.38	0.10–1.59	
12-months follow-up	0.84 *	0.37	0.13–1.56	
VOICES vs. HEALTHY	−0.6 *	0.31	−1.29–0.08	
Adequate knowledge of condom use				0.08
6-months follow-up	1.11 *	0.45	0.23–1.99	
12-months follow-up	2.74 ***	0.41	1.94–3.55	
VOICES vs. HEALTHY	−0.59	0.36	−1.29–0.10	
*Behavioral Factors*				
Condom use				
6-months follow-up	0.41 **	0.14	0.14–0.69	0.01
12-months follow-up	0.37 **	0.12	0.13–0.62	
VOICES vs. HEALTHY	0.08	0.22	−0.35–0.52	
Female condom use				
6-months follow-up	0.62 *	0.30	0.05–1.20	0.01
12-months follow-up	0.75 **	0.29	0.19–1.32	
VOICES vs. HEALTHY	0.13	0.24	−1.16–0.71	
HIV testing				0.03
Changes from before intervention (baseline) to during intervention (6-months and 12-months follow-up)	0.77 ***	0.21	0.37–1.18	
VOICES vs. HEALTHY	−0.34	0.21	−0.75–0.07	
*Communication Factors*				
Talk with friends about HIV prevention				0.07
6-months follow-up	1.11 ***	0.25	0.63–1.59	
12-months follow-up	1.51 ***	0.26	1.01–2.02	
Intervention VOICES	0.40	0.21	−0.01–0.81	
Intention to negotiate safe sex with male partner				
6-months follow-up	0.15 *	0.07	0.01–0.29	0.01
12-months follow-up	0.05	0.07	−0.09–0.20	
VOICES vs. HEALTHY	0.05	0.06	−0.07–0.16	

* *p* < 0.05, ** *p* < 0.01, *** *p* < 0.001.

## Supplementary Materials

The following are available online at https://www.mdpi.com/1660-4601/16/22/4530/s1, Table S1: Original and adapted core components of VOICES, Figure S2: Flow diagram of participants through intervention arms, South Florida, 2015–2017.

## References

[1] Centers for Disease Control and Prevention (2015). CDC Fact Sheet: HIV among Latinos.

[2] Centers for Disease Control and Prevention HIV and Hispanics/Latinos. https://www.cdc.gov/hiv/group/racialethnic/hispaniclatinos/index.html.

[3] Centers for Disease Control and Prevention (2017). HIV among Latinos: A Snapshot.

[4] Henao-Martinez A., Castillo-Mancilla J. (2013). The Hispanic HIV epidemic. Curr. Infect. Dis. Rep..

[5] Shriver M. (2014). The Shriver Report: A Woman’s Nation Pushes Back from the Brink.

[6] Snyder T., de Brey C., Dillow S. (2016). Digest of Education Statistics 2014 NCES 2016-006.

[7] Kanamori M., De La Rosa M., Diez S., Weissman J., Trepka M.J., Sneij A., Schmidt P., Rojas P. (2016). A brief report: Lessons learned and preliminary findings of Progreso en Salud, an HIV risk reduction intervention for Latina seasonal farmworkers. Int. J. Environ. Res. Public Health.

[8] McCullagh M.C., Sanon M.-A., Foley J.G. (2015). Cultural health practices of migrant seasonal farmworkers. J. Cult. Divers..

[9] National Center for Farmworker Health (2017). Farmworker Fact. Sheet.

[10] National Center for Farmworker Health (2017). HIV/AIDS Agricultural Worker Factsheet.

[11] Centers for Disease Control and Prevention (2017). HIV Surveillance Report: Diagnoses of HIV Infection in the United States and Dependent Areas. https://www.cdc.gov/hiv/pdf/library/reports/surveillance/cdc-hiv-surveillance-report-2017-vol-29.pdf.

[12] Florida Department of Health, Bureau of Communicable Diseases, HIV/AIDS Section (2018). Epidemiological Profile for Area 11, Miami-Dade County. http://miamidade.floridahealth.gov/programs-and-services/infectious-disease-services/hiv-aids-services/_documents/10-26-18-update-HIV-Surveillance/_documents/FS-2017-MIAMI-DADE.pdf.

[13] Florida Department of Health Miami-Dade County (2016). HIV/AIDS Surveillance Electronic HIV/AIDS Reporting System [e-HARS]. In Frozen database on June 2016 ed. http://miamidade.floridahealth.gov/programs-and-services/infectious-disease-services/hiv-aids-services/_documents/1-6-17-hiv-aids-surveillance-monthly-report/_documents/hiv-surveillance-monthly-report-2016-11.pdf.

[14] Cianelli R., Villegas N. (2016). Social Determinants of Health for HIV among Hispanic Women.

[15] Acevedo V. (2008). Cultural competence in a group intervention designed for Latino patients living with HIV/AIDS. Health Soc. Work.

[16] Ghosh D., Krishnan A., Gibson B., Brown S.-E., Latkin C.A., Altice F.L. (2017). Social network strategies to address HIV prevention and treatment continuum of care among at-risk and HIV-infected substance users: A systematic scoping review. AIDS Behav..

[17] Schulz K.F., Altman D.G., Moher D. (2010). CONSORT 2010 statement: Updated guidelines for reporting parallel group randomised trials. BMC Med..

[18] Añez L.M., Paris M., Bedregal L.E., Davidson L., Grilo C.M. (2005). Application of cultural constructs in the care of first generation Latino clients in a community mental health setting. J. Psychiatr. Pract..

[19] Comas-Diaz L. (2006). Latino healing: The integration of ethnic psychology into psychotherapy. Psychother. Theory Res. Pract. Train..

[20] Damschroder L.J., Lowery J.C. (2013). Evaluation of a large-scale weight management program using the consolidated framework for implementation research (CFIR). Implement. Sci..

[21] Peterson-Iyer K. (2008). Culturally Competent Care for Latino Patients: Introduction.

[22] Heckathorn D.D. (2011). Comment: Snowball versus respondent-driven sampling. Sociol. Methodol..

[23] Farquhar S., de Jesus Gonzalez C., Hall J., Samples J., Ventura S., Sanchez V., Shadbeh N. (2014). Recruiting and retaining indigenous farmworker participants. J. Immigr. Minor. Health.

[24] Ajzen I., Fishbein M. (1980). Understanding Attitudes and Predicting Social Behaviour.

[25] Neumann M.S., O’Donnell L., San Doval A., Schillinger J., Blank S., Ortiz-Rios E., Garcia T., O’Donnell C.R. (2011). Effectiveness of the VOICES/VOCES sexually transmitted disease/human immunodeficiency virus prevention intervention when administered by health department staff: Does it work in the “real world”?. Sex. Transm. Dis..

[26] Fisher H.H., Patel-Larson A., Green K., Shapatava E., Uhl G., Kalayil E., Moore A., Williams W., Chen B. (2011). Evaluation of an HIV prevention intervention for African Americans and Hispanics: Findings from the VOICES/VOCES Community-based Organization Behavioral Outcomes Project. AIDS Behav..

[27] Hamdallah M., Vargo S., Herrera J. (2006). The VOICES/VOCES success story: Effective strategies for training, technical assistance and community-based organization implementation. AIDS Educ. Prev..

[28] O’Donnell L.N., Doval A.S., Duran R., O’Donnell C. (1995). Video-based sexually transmitted disease patient education: Its impact on condom acquisition. Am. J. Public Health.

[29] O’Donnell L., Stueve A., Joseph H.A., Flores S. (2014). Adapting the VOICES HIV behavioral intervention for Latino men who have sex with men. AIDS Behav..

[30] Stallworth J.M., Andía J.F., Burgess R., Alvarez M.E., Collins C. (2009). Diffusion of effective behavioral interventions and Hispanic/Latino populations. AIDS Educ. Prev..

[31] Kanamori M., De La Rosa M., Diez S.L., Weissman J., Trepka M.J., Sneij A., Schmidt P., Rojas P. (2017). Progreso en Salud, An HIV risk reduction intervention for Latina seasonal farmworkers: Preliminary findings. Annals of Behavioral Medicine.

[32] Bryan A.D., Aiken L.S., West S.G. (1996). Increasing condom use: Evaluation of a theory-based intervention to prevent sexually transmitted diseases in young women. Health Psychol..

[33] Zeger S.L., Liang K.-Y., Albert P.S. (1988). Models for longitudinal data: A generalized estimating equation approach. Biometrics.

[34] Hubbard A.E., Ahern J., Fleischer N.L., Van der Laan M., Satariano S.A., Jewell N., Bruckner T., Satariano W.A. (2010). To GEE or not to GEE: Comparing population average and mixed models for estimating the associations between neighborhood risk factors and health. Epidemiology.

[35] Wang K., Brown K., Shen S.Y., Tucker J. (2011). Social network-based interventions to promote condom use: A systematic review. AIDS Behav..

[36] Dunning T., Freedman D., Outhwaite W., Turner S.P. (2007). Modeling selection effects. The Sage Handbook of Social Science Methodology.

[37] Christakis N.A., Fowler J.H. (2011). Connected: The Surprising Power of Our Social Networks and How They Shape Our Lives—How Your Friends’ Friends’ Friends Affect Everything You Feel, Think, and Do.

[38] Fowler J.H., Christakis N.A. (2010). Cooperative behavior cascades in human social networks. Proc. Natl. Acad. Sci. USA.

[39] Valente T., Ritt-Olson A., Stacy A., Unger J., Okamoto J., Sussman S. (2007). Peer acceleration: Effects of a social network tailored substance abuse prevention program among high-risk adolescents. Addiction.

[40] Rand D., Arbesman S., Christakis N. (2011). Dynamic social networks promote cooperation in experiments with humans. Proc. Natl. Acad. Sci. USA.

[41] Banerjee A., Chandrasekhar A.G., Duflo E., Jackson M.O. (2013). The diffusion of microfinance. Science.

[42] Merzel C., D’Afflitti J. (2003). Reconsidering community-based health promotion: Promise, performance, and potential. Am. J. Public Health.

[43] Stephens A.J., Tchetgen E.J.T., De Gruttola V. (2012). Augmented GEE for improving efficiency and validity of estimation in cluster randomized trials by leveraging cluster-and individual-level covariates. Stat. Med..

[44] Panageas K.S., Schrag D., Russell Localio A., Venkatraman E., Begg C.B. (2007). Properties of analysis methods that account for clustering in volume–outcome studies when the primary predictor is cluster size. Stat. Med..

[45] Hussey M.A., Hughes J.P. (2007). Design and analysis of stepped wedge cluster randomized trials. Contemp. Clin. Trials.

[46] Food and Drug Administration (2012). Truvada Approved to Reduce the Risk of Sexually Transmitted HIV in People Who Are Not Infected with the Virus. https://www.fda.gov/media/83604/download.

[47] Thigpen M.C., Kebaabetswe P.M., Paxton L.A., Smith D.K., Rose C.E., Segolodi T.M., Henderson F.L., Pathak S.R., Soud F.A., Chillag K.L. (2012). Antiretroviral preexposure prophylaxis for heterosexual HIV transmission in Botswana. N. Engl. J. Med..

[48] Baeten J.M., Donnell D., Ndase P., Mugo N.R., Campbell J.D., Wangisi J., Tappero J.W., Bukusi E.A., Cohen C.R., Katabira E. (2012). Antiretroviral prophylaxis for HIV prevention in heterosexual men and women. N. Engl. J. Med..

[49] Grant R.M., Lama J.R., Anderson P.L., McMahan V., Liu A.Y., Vargas L., Goicochea P., Casapía M., Guanira-Carranza J.V., Ramirez-Cardich M.E. (2010). Preexposure chemoprophylaxis for HIV prevention in men who have sex with men. N. Engl. J. Med..

[50] Anderson P.L., Glidden D.V., Liu A., Buchbinder S., Lama J.R., Guanira J.V., McMahan V., Bushman L.R., Casapía M., Montoya-Herrera O. (2012). Emtricitabine-tenofovir concentrations and pre-exposure prophylaxis efficacy in men who have sex with men. Sci. Transl. Med..

[51] Aaron E., Blum C., Seidman D., Hoyt M.J., Simone J., Sullivan M., Smith D.K. (2018). Optimizing delivery of HIV preexposure prophylaxis for women in the United States. AIDS Patient Care STDs.

[52] Walters S.M., Rivera A.V., Starbuck L., Reilly K.H., Boldon N., Anderson B.J., Braunstein S. (2017). Differences in awareness of pre-exposure prophylaxis and post-exposure prophylaxis among groups at-risk for HIV in New York State: New York City and Long Island, NY, 2011–2013. JAIDS J. Acquir. Immune Defic. Syndr..

[53] Eaton L.A., Matthews D.D., Driffin D.D., Bukowski L., Wilson P.A., Stall R.D., Team P.S. (2017). A multi-US city assessment of awareness and uptake of pre-exposure prophylaxis (PrEP) for HIV prevention among Black men and transgender women who have sex with men. Prev. Sci..

[54] Flash C.A., Dale S.K., Krakower D.S. (2017). Pre-exposure prophylaxis for HIV prevention in women: Current perspectives. Int. J. Women’s Health.

