# Supplementary material for: Progreso en Salud: Findings from Two Adapted Social Network HIV Risk Reduction Interventions for Latina Seasonal Workers
Source: Int J Environ Res Public Health. 2019 Nov 15;16(22):4530. doi: 10.3390/ijerph16224530 (PMC6888294; doi:10.3390/ijerph16224530)
Supplement: Supplementary file 1 [file ijerph-16-04530-s001.pdf]

Progreso en Salud: Findings from Two Adapted Social Network HIV Risk Reduction Interventions for Latina Seasonal Workers

Supplementary Materials:

Table S1. Original and adapted core components of VOICES.

Original VOICES	Adapted VOICES
Component 1	
Facilitators present a culturally specific video portraying condom use and negotiation. The actors in the video present information on HIV/STI* risk behavior and model condom use and negotiation.	The video was a *telenovela*, a Latino soap opera format, shown to workers during lunch or dinner. While participants were eating, the Project Coordinator and Lay Health Advisor encouraged participants to discuss the scenes and various ways to handle the situations presented in the *telenovela* using a standardized protocol. These discussions followed a consistent format, but the adaptation lay in the tailoring of the content to address the concerns and experiences of each group. This approach was used to resemble a conversation at a social or family event and thus incorporated the value of *personalismo*.
Component 2	
Condom negotiation is role-played, practiced, and discussed. Facilitators ask specific questions about the characters and events depicted in the video, and then encourage clients to relate these situations to their own lives. Facilitators provide information and correct misinformation in regards to the *telenovela.* Facilitators do not discuss condom options at three and nine months post intervention.	Condom negotiation was role-played, practiced, and discussed by participants after instruction on how to use dolls to enact a real-life scenario. The hypothetical scene began with the couple on a romantic date, later becoming physically intimate at his/her house. The woman suggested using a condom before proceeding with sexual intimacy and ultimately penetrative sex; however, the male refused to use a condom. All participants were then instructed to discuss ways to negotiate with the man and to ultimately convince him to use a condom.<br>Visual materials were included during discussions to facilitate participants' understanding of the messages presented in the video. The Project Coordinator and Lay Health Advisor used a series of flip-chart posters with large graphical representations to provide information and promote discussion regarding HIV and STIs including sex and drug-related modes of transmission, HIV statistics, and ways to prevent HIV/STI infection. The content of these flip-chart posters mirrored the messages included in the original VOICES strategy. Adequate interpretation of the messages and cultural acceptance of the figures were pilot tested with members of the community.<br>We provided Latina Leaders with additional instructions on how to promote interactions and conversations about HIV prevention within their group of friends. Three months and nine months after the intervention session, Latina Leaders met with the individuals who participated in their groups to discuss HIV/STI prevention, either as a group, in a single session, or via individual home visits depending on the participants' availability. The Latina Leaders were given three HIV/STI informational pamphlets in Spanish to guide the conversations each time. These pamphlets were also distributed to each member of their groups.
Component 3	

Facilitators use a poster to show features of various condom brands in English and Spanish. Participants practice correct condom placement. The original VOICES kit includes only one penis model. There are no assessments to assess participant understanding from components 1–3.	Facilitators used a poster to show features of various condom brands in English and Spanish. We added information and demonstrations on how to use female condoms because the original VOICES intervention and materials included only male condoms. Participants practiced correct condom placement. We included additional penis models in our intervention. All participants had the opportunity to simultaneously practice correct condom placement on the penis models. Facilitators conducted informal assessments using a list of messages to examine if participants understand content from components 1–3.
Component 4	
At the end of the session, participants are given samples of the types of condoms they have identified as best meeting their needs.	At the end of the session, participants were given samples of the types of condoms they identified as best meeting their needs. We also offered female condoms.

*STI: Sexually transmitted infection.

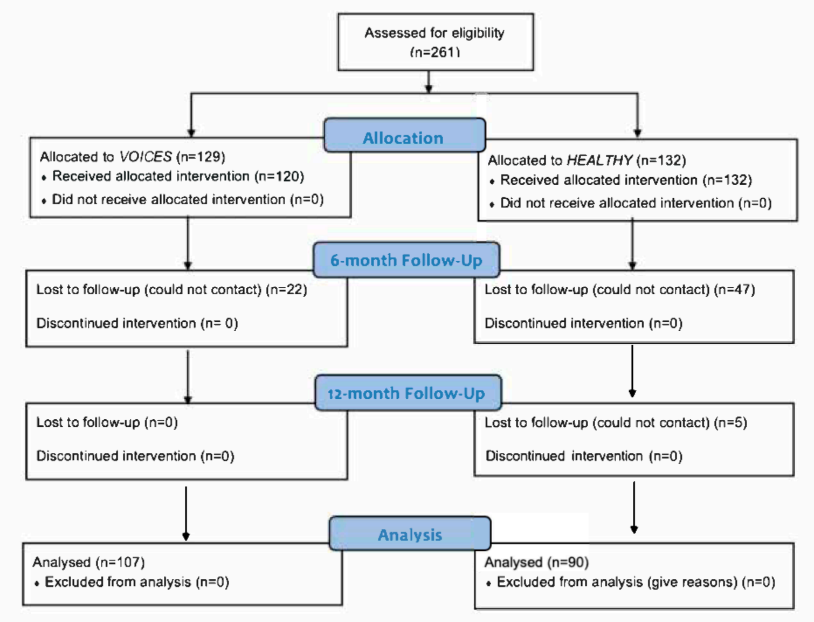

Figure S2. Flow diagram of participants through intervention arms, South Florida, 2015–2017.
